# Case Report: A hepatic lesion is not always metastasis: synchronous rectal adenocarcinoma and primary hepatocellular carcinoma followed by a benign necrotic liver nodule

**DOI:** 10.3389/fmed.2026.1921118

**Published:** 2026-08-20

**Authors:** Huiying Liu, Jing Gao, Weiting Zhang, Xiaoxia Kou, Huafei Li, Jinrong Qiu

**Affiliations:** 1 Department of Cancer Biotherapy, The Third Affiliated Hospital of Navy Medical University, Shanghai, China; 2 School of Lifesciences, Shanghai University, Shanghai, China; 3 South Taihu Hospital Affiliated to Huzhou Collage, Huzhou, China

**Keywords:** colorectal cancer, colorectal liver metastasis, hepatic necrotic nodule, hepatocellular carcinoma, multiple primary malignancy

## Abstract

**Background:**

Liver lesions identified during postoperative surveillance of colorectal cancer are commonly presumed to represent colorectal liver metastases. However, patients with chronic liver disease may develop additional primary hepatic malignancies, creating significant diagnostic and therapeutic challenges. We report a rare case of rectal adenocarcinoma followed by pathologically confirmed primary HCC and a subsequent benign hepatic necrotic nodule, both initially suspected to represent metastatic or recurrent disease.

**Case description:**

A 56-year-old man with a 20-year history of chronic hepatitis B was diagnosed with rectal adenocarcinoma and underwent laparoscopic Low Anterior Resection. Seven months later, a solitary hepatic lesion was identified during surveillance. Although colorectal liver metastasis was initially suspected, elevated alpha-fetoprotein levels and characteristic imaging findings suggested primary HCC. Right hepatectomy confirmed moderately to poorly differentiated HCC with microvascular invasion. Five months after adjuvant transarterial chemoembolization, another hepatic lesion was detected and considered highly suspicious for recurrence. Repeat resection was performed, but pathology revealed only necrosis and fibrosis without viable tumor cells, confirming a benign necrotic hepatic nodule. No recurrence or metastasis was observed during follow-up.

**Conclusion:**

This case highlights the diagnostic complexity of hepatic lesions arising in patients with colorectal cancer and chronic hepatitis B infection. Not all hepatic lesions represent metastatic disease, and accurate diagnosis requires integration of clinical history, imaging findings, tumor biomarkers, and histopathological confirmation.

## 1 Introduction

Colorectal cancer (CRC) is among the most common malignancies worldwide and remains a major cause of cancer-related mortality. The liver is the predominant site of distant metastasis, with approximately 40–50% of patients developing hepatic involvement during the course of their disease (1). Consequently, newly identified liver lesions in patients with a history of CRC are frequently presumed to represent colorectal liver metastases (CLM) (2).

Despite the high prevalence of CLM, hepatic lesions identified during surveillance are not invariably metastatic (3). Patients with chronic liver diseases, particularly chronic hepatitis B virus (HBV) infection, remain at increased risk of developing hepatocellular carcinoma (HCC) (4). In these patients, distinguishing primary HCC from CLM is clinically essential because treatment strategies, surgical indications, and long-term prognoses differ substantially.

Although advances in contrast-enhanced magnetic resonance imaging (MRI), positron emission tomography-computed tomography (PET-CT), and radiomics-based approaches have improved diagnostic accuracy, overlap in imaging characteristics may still create significant diagnostic uncertainty. Histopathological examination therefore remains the gold standard for definitive diagnosis. In addition, benign hepatic lesions may occasionally mimic malignant tumors radiologically, further complicating clinical decision-making (5). The coexistence of colorectal cancer, chronic hepatitis B infection, and multiple hepatic lesions presents a unique diagnostic challenge that requires careful multidisciplinary evaluation (6).

Here, we report a rare case of rectal adenocarcinoma followed by pathologically confirmed primary HCC and a subsequent benign necrotic hepatic nodule. This case highlights the limitations of presumptive radiological diagnosis and emphasizes the indispensable role of pathological confirmation in the management of complex hepatic lesions.

## 2 Case presentation

A 56-year-old Chinese man presented to Binhai Kangda Hospital, Jiangsu Province, China, with a two-year history of altered bowel habits accompanied by intermittent hematochezia. His past medical history was significant for chronic hepatitis B virus infection for more than 20 years. Notably, he had not received antiviral therapy at the initial diagnosis of rectal cancer or primary HCC; oral antiviral therapy was initiated later in February 2023. No family history of colorectal cancer or hepatocellular carcinoma was reported.

Initial colonoscopic examination performed on June 15, 2022 revealed multiple colorectal polyps and an irregular friable rectal mass located approximately 5 cm from the anal verge. The patient was subsequently referred to Nanjing Hospital of Traditional Chinese Medicine for further evaluation. Repeat colonoscopy confirmed a 3.0 × 3.0 cm rectal lesion. Histological examination demonstrated high-grade intraepithelial neoplasia with invasive carcinoma in the rectum, whereas the colonic polyps showed villous-tubular adenomas with low-grade dysplasia.

Preoperative staging investigations revealed no evidence of distant metastatic disease. Serum tumor markers, including AFP (1.88 ng/mL), CEA (1.24 ng/mL), and CA19-9 (11.44 U/mL), were all within normal ranges. The patient subsequently underwent laparoscopic Low Anterior Resection 1 week later.

**Figure 1:**
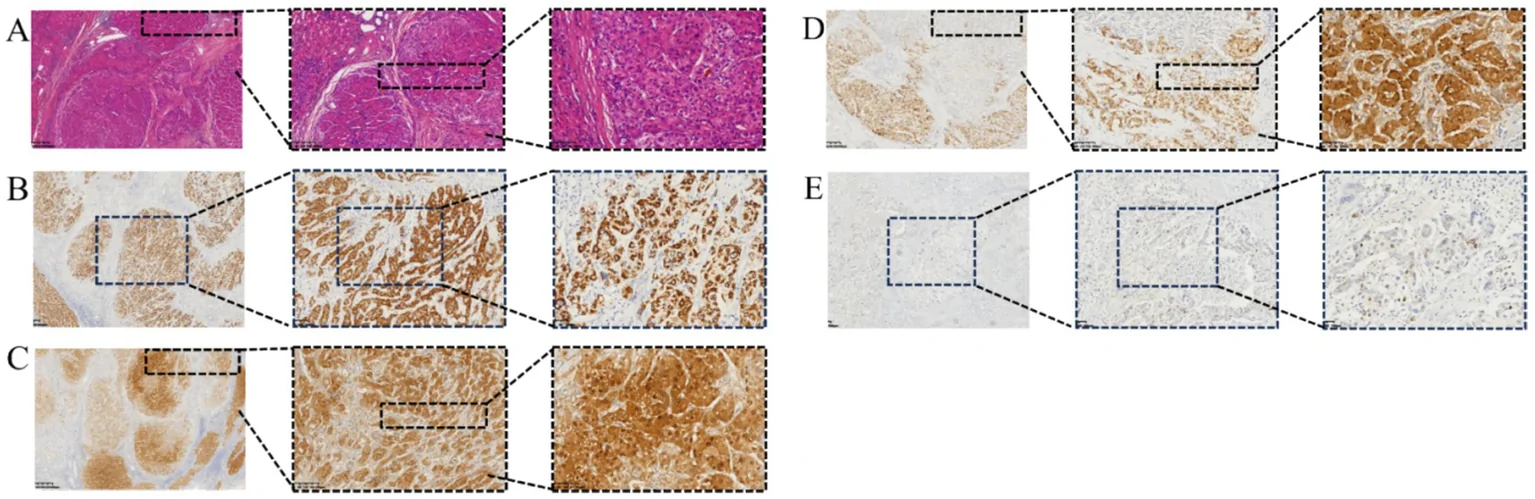
**(A–E)** HE, Hep-1, Arginase, GPC3, and KI67 staining of rectal cancer tissues (left panel×40, middle panel×100, right panel×200).

Histopathological examination of the resected specimen demonstrated moderately differentiated rectal adenocarcinoma with vascular tumor emboli, perineural invasion, and prominent tumor budding. Achieving an R0 resection, both the proximal and distal bowel margins as well as the circumferential resection margin (CRM) were negative, with no tumor involvement. Immunohistochemical analysis revealed preserved mismatch repair protein expression, wild-type p53 expression, and a Ki-67 labeling index of approximately 60% within hotspot regions. Representative histological and immunohistochemical findings are presented in Figures 1A–E.

At seven months of postoperative surveillance, routine computed tomography identified a low-density lesion within the right hepatic lobe, raising concern for malignant disease. Given the patient’s history of rectal cancer, colorectal liver metastasis was initially considered. However, because of the patient’s longstanding hepatitis B infection, additional investigations were undertaken.

**Figure 2:**
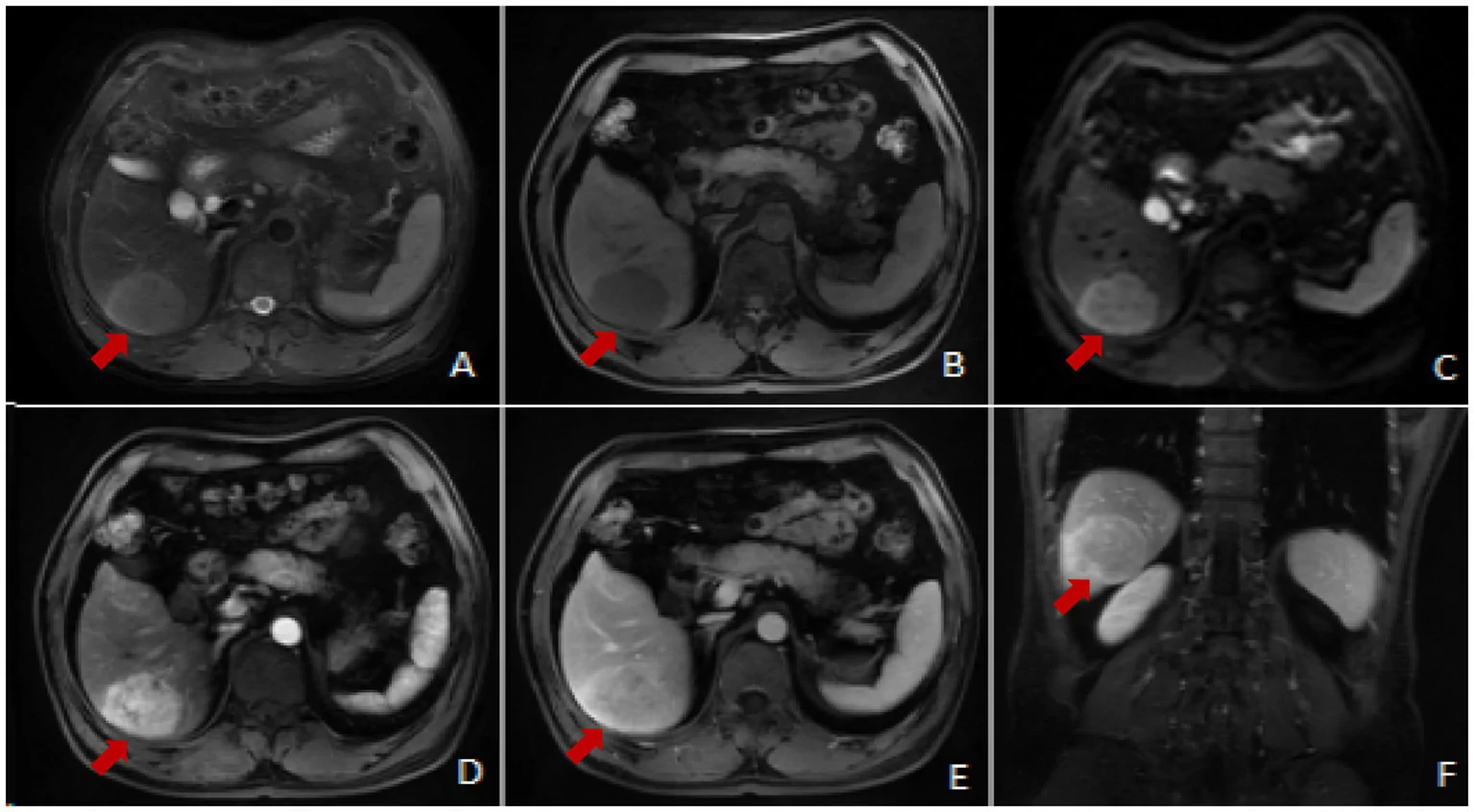
**(A)** MR T2-weighted imaging (T2WI) shows slightly high signal intensity in the lesion of the right posterior hepatic lobe. **(B)** T1-weighted imaging (T1WI) shows low signal intensity. **(C)** Diffusion-weighted imaging (DWI, *b* = 600 s/mm) shows high signal intensity. **(D)** Arterial phase shows significant enhancement. **(E)** Portal venous phase shows clear delineation of the lesion. **(F)** Coronal delayed phase shows ring-like capsule enhancement.

Contrast-enhanced MRI demonstrated a 5.2 × 4.0 cm lesion located in the posterior segment of the right hepatic lobe. The lesion exhibited low signal intensity on T1-weighted imaging, mildly increased signal intensity on T2-weighted imaging, restricted diffusion, marked arterial phase hyperenhancement, and delayed washout with capsule-like enhancement, imaging characteristics highly suggestive of hepatocellular carcinoma (Figures 2A–F).

**Figure 3:**
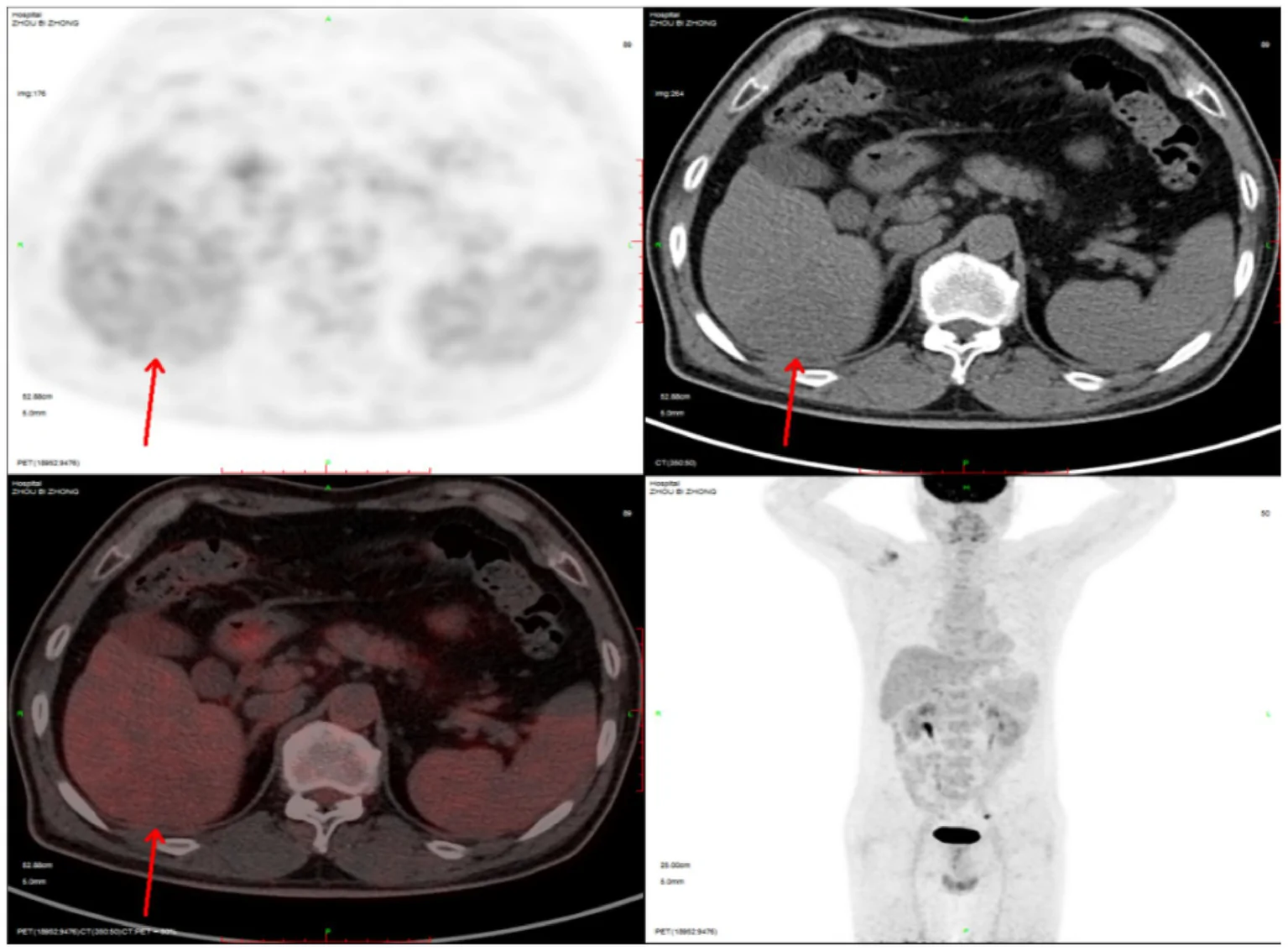
PET/CT: non-contrast CT reveals a nodular, mildly hypodense lesion in the posterior segment of the right lobe of the liver, with poorly defined borders. Mildly increased FDG uptake is noted (SUVmax = 3.1). The lesion measures approximately 5.8 × 3.7 cm.

Upon admission to Shanghai Eastern Hepatobiliary Surgery Hospital, comprehensive evaluation of tumor biomarkers was performed to differentiate potential malignancies. Laboratory evaluation demonstrated markedly elevated AFP levels (128.8 ng/mL) and abnormal prothrombin concentrations (345 mAU/mL), whereas other markers such as carcinoembryonic antigen (CEA) and CA19-9 remained within normal ranges. Serological testing confirmed chronic hepatitis B infection. Whole-body PET-CT demonstrated a mildly hypermetabolic hepatic lesion measuring approximately 5.8 × 3.7 cm with a maximum standardized uptake value (SUVmax) of 3.1 and no evidence of extrahepatic disease (Figure 3).

The coexistence of rectal cancer and chronic hepatitis B infection created a significant diagnostic dilemma because both colorectal liver metastasis and primary hepatocellular carcinoma represented plausible explanations for the hepatic lesion. Following multidisciplinary discussion, surgical resection was recommended for definitive diagnosis and treatment.

**Figure 4:**
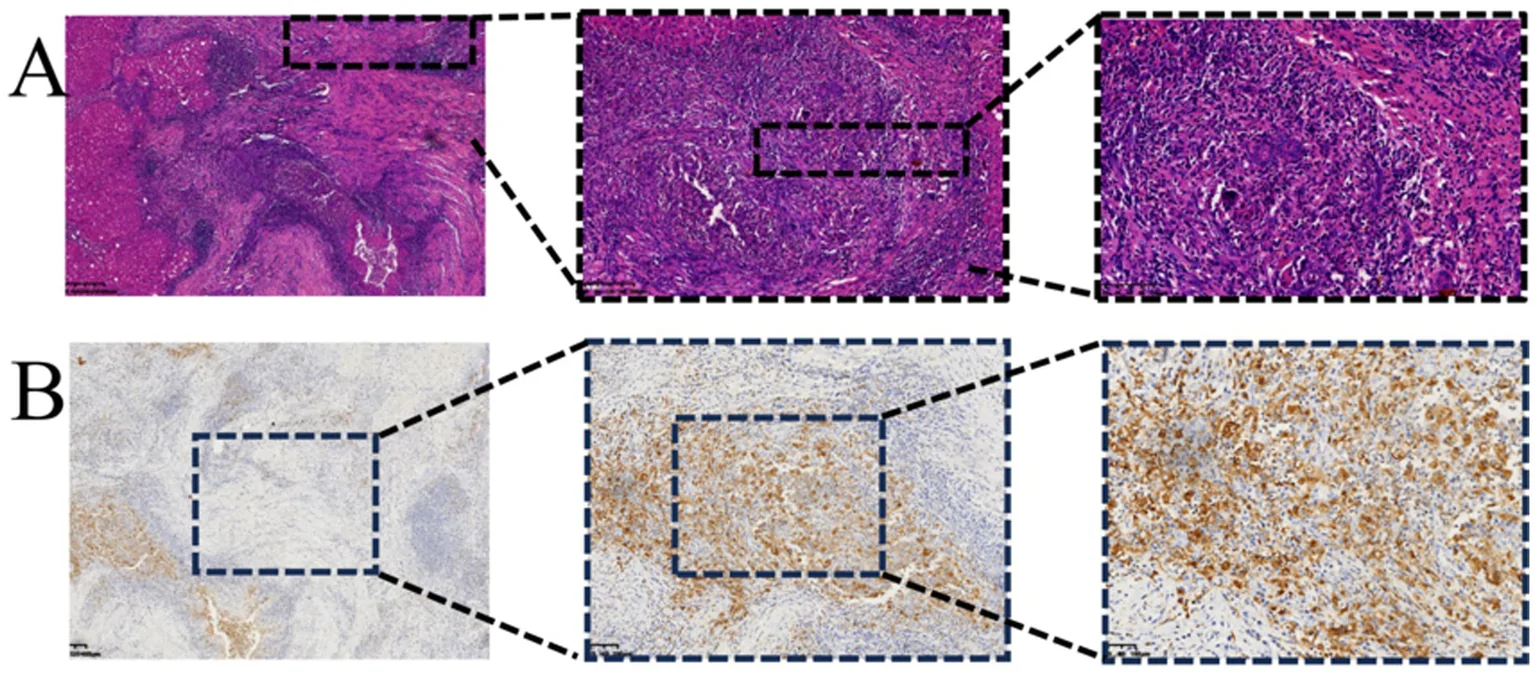
**(A)** HE staining of liver cancer tissues (left panel×40, middle panel×100, right panel×200). **(B)** CD68 staining of liver cancer tissues (left panel×40, middle panel×100, right panel×200).

Following multidisciplinary discussion, surgical resection was recommended, and the patient underwent right hepatectomy the following month. Intraoperatively, a solitary tumor measuring approximately 5 cm in diameter was identified within the right posterior hepatic lobe. Histopathological examination demonstrated trabecular hepatocellular carcinoma with poor differentiation (grade III–IV) and microvascular invasion. Background liver tissue showed chronic hepatitis with mild steatosis. Representative pathological findings are shown in Figures 4A,B.

Because of the presence of microvascular invasion, adjuvant transarterial chemoembolization (TACE) was performed approximately 1 month postoperatively. The procedure was completed without complications.

**Figure 5:**
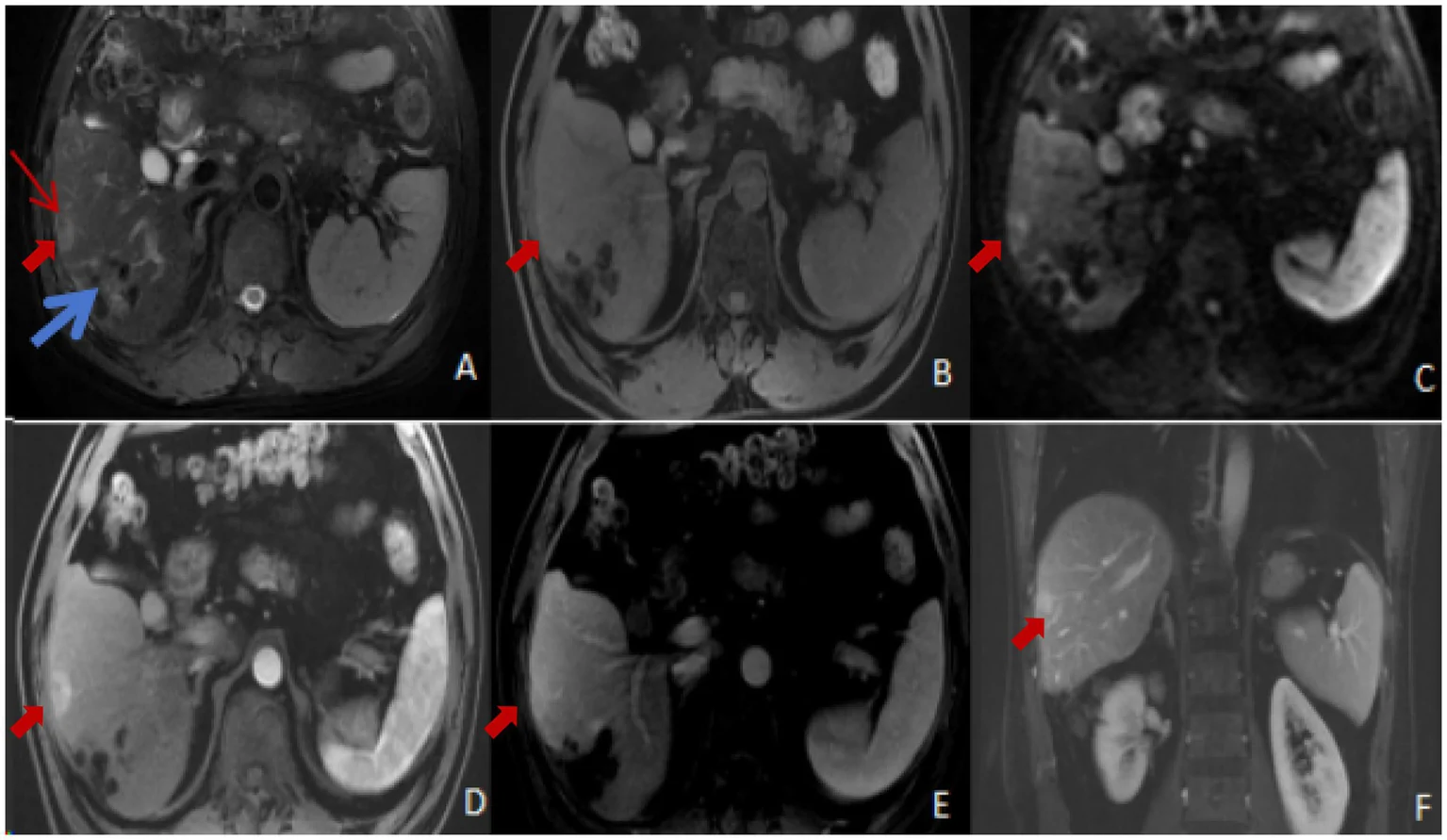
Enhanced MRI of the upper abdomen: **(A)** MR T2WI shows slightly high, uneven signal in the subcapsular area of the right anterior lobe, lower segment, near the surgical site. **(B)** T1WI is unclear. **(C)** DWI (*b* = 600 s/mm) shows high signal. **(D)** Significant enhancement in the arterial phase. **(E)** Mild enhancement in the portal venous phase. **(F)** Coronal view shows sustained enhancement in the delayed phase, higher than the liver parenchyma signal.

Five months later, surveillance MRI revealed a new lesion located adjacent to the previous surgical site. The lesion demonstrated increased signal intensity on diffusion-weighted imaging, arterial enhancement, and persistent delayed enhancement, findings concerning for recurrent HCC or colorectal liver metastasis (Figures 5A–F). Given the patient’s oncologic history, recurrent malignancy could not be excluded. Serial laboratory monitoring demonstrated generally stable tumor marker levels throughout follow-up. Liver function tests showed transient elevations of ALT and AST, with peak values observed in July 2023, followed by gradual improvement. GGT and ALP remained relatively stable. Hematological parameters, including white blood cell count, red blood cell count, and hemoglobin levels, were maintained within normal ranges.

**Figure 6:**
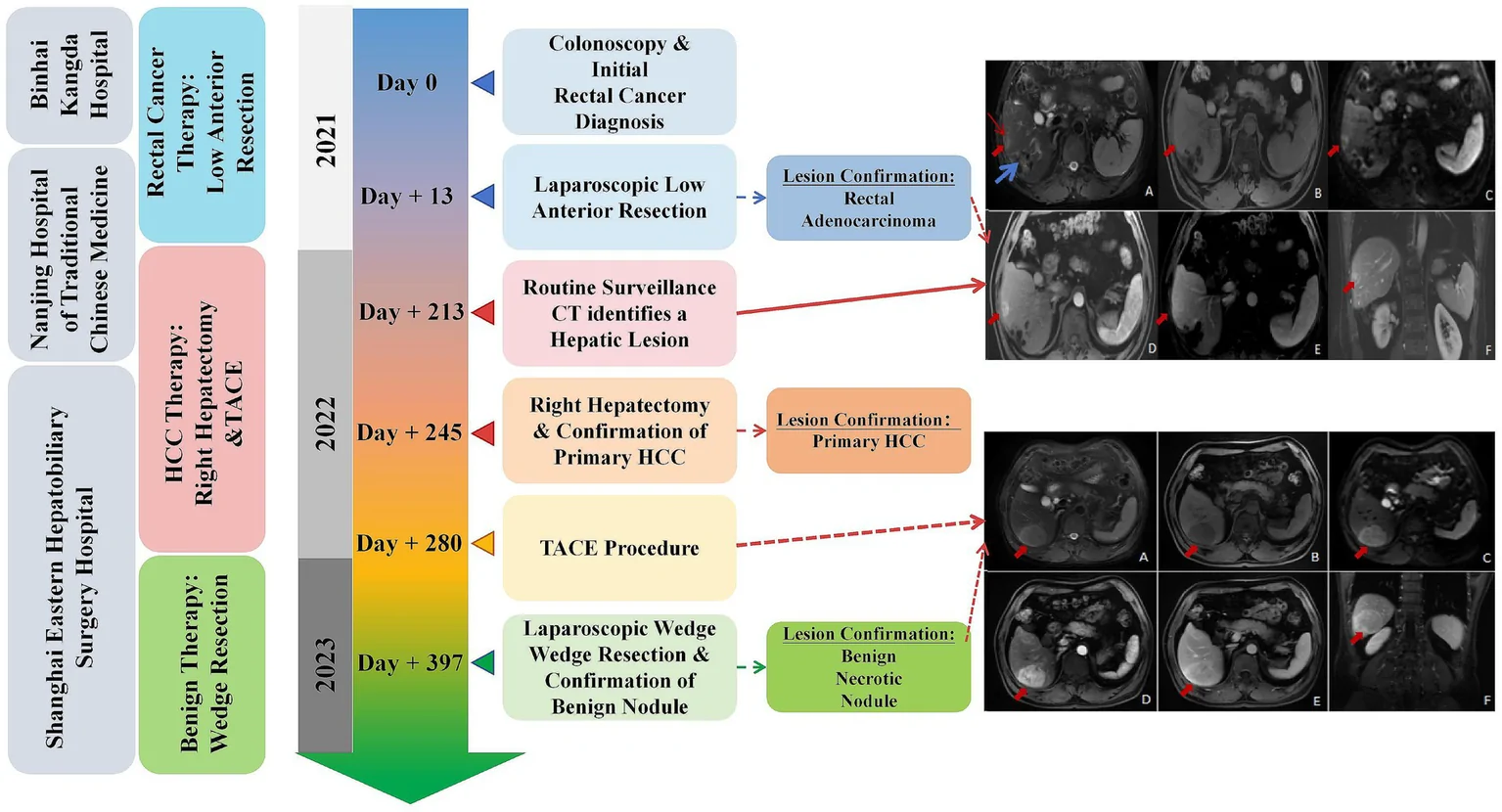
Timeline of the clinical course for the patient with rectal adenocarcinoma, followed by primary hepatocellular carcinoma (HCC) and a benign necrotic liver nodule. Date markers are in relative days (Day 0 = initial rectal cancer diagnosis). Right selected MRI frames. Figures 2A–D shows characteristic findings of primary HCC (Day +213). Figures 5A–D shows characteristic findings of a benign necrotic hepatic nodule (Day +397). (red arrows point to the lesion).

The patient subsequently underwent laparoscopic wedge resection in July 2023. Unexpectedly, pathological examination demonstrated extensive necrosis and fibrosis without viable malignant cells, confirming an R0 resection with negative surgical margins. Immunohistochemical analysis showed no evidence of recurrent carcinoma, confirming the diagnosis of a benign necrotic hepatic nodule rather than recurrent HCC or metastatic rectal cancer (Figure 6).

## 3 Discussion

This case illustrates several important diagnostic and therapeutic challenges encountered during the management of hepatic lesions in patients with colorectal cancer. The liver is the most common site of distant metastasis in colorectal cancer, and hepatic lesions identified during postoperative surveillance are frequently presumed to represent metastatic disease (7). Consequently, hepatic lesions detected during postoperative surveillance are often presumed to represent colorectal liver metastasis (CLM) (8). While this assumption is reasonable in many clinical settings, it may result in diagnostic anchoring and potentially inappropriate management if alternative etiologies are not adequately considered. The present patient had chronic hepatitis B infection, which substantially increased his risk of developing primary hepatocellular carcinoma. Chronic hepatitis B virus (HBV) infection remains one of the strongest established risk factors for HCC worldwide because persistent viral infection promotes chronic inflammation, fibrosis, genomic instability, and hepatocarcinogenesis (9, 10). Current international guidelines therefore recommend careful surveillance for HCC in patients with chronic HBV infection, even in the absence of advanced cirrhosis (10). The coexistence of colorectal cancer and chronic liver disease created a unique diagnostic dilemma because both CLM and HCC were plausible explanations for the first hepatic lesion. Although MRI findings and elevated AFP levels favored HCC, imaging findings alone could not completely exclude metastasis. This highlights the importance of considering the patient’s underlying liver disease rather than interpreting hepatic lesions solely in the context of a previous colorectal malignancy. Definitive diagnosis required pathological confirmation.

Another important consideration is that radiological enhancement patterns, although highly informative, are not entirely disease-specific. Multiphasic contrast-enhanced CT and MRI play central roles in differentiating HCC from metastatic liver tumors, with HCC typically demonstrating arterial phase hyperenhancement followed by washout during the portal venous or delayed phases. In contrast, colorectal liver metastases more commonly exhibit peripheral rim enhancement and delayed progressive enhancement. Nevertheless, substantial overlap exists between these imaging characteristics, particularly in patients with chronic liver disease or atypical lesions (11, 12). Furthermore, several benign hepatic lesions—including inflammatory pseudotumors, regenerative nodules, focal necrosis, and organizing hematomas—may also demonstrate enhancement patterns that mimic malignant tumors (13, 14). Therefore, imaging findings should always be interpreted in conjunction with clinical history, laboratory biomarkers, and individual risk factors.

Several similar cases have been reported in the literature. Karass et al. described metastatic colorectal cancer occurring in a cirrhotic liver with synchronous hepatocellular carcinoma, emphasizing the difficulty of distinguishing between metastatic and primary hepatic malignancies (15). Likewise, Alwabel and colleagues reported colorectal cancer accompanied by primary liver cancer, highlighting the importance of comprehensive diagnostic evaluation (16). Similar to these reports, our patient required integration of clinical history, imaging findings, tumor biomarkers, and pathology to establish the correct diagnosis.

An additional unique aspect of the present case was the occurrence of a second hepatic lesion that ultimately proved to be a benign necrotic nodule. Unlike previously reported cases in which subsequent lesions represented recurrent or metastatic malignancy (17), pathological examination in our patient revealed only fibrosis and necrosis without viable tumor cells. This finding further demonstrates that not all enhancing hepatic lesions in oncologic patients represent active cancer.

The major strength of this report is the availability of pathological confirmation for both hepatic lesions. Histological verification allowed accurate differentiation among metastatic disease, primary hepatocellular carcinoma, and benign pathology, thereby avoiding diagnostic uncertainty. Current international guidelines recognize histopathological confirmation as the diagnostic gold standard whenever imaging findings are atypical, conflicting, or insufficiently characteristic for a definitive diagnosis (9, 10). Although biopsy is not routinely required for lesions with classical imaging characteristics of HCC, tissue diagnosis remains invaluable when multiple competing diagnoses exist, as illustrated in this case. In addition, the patient underwent long-term follow-up, allowing assessment of clinical outcomes after treatment.

Several limitations should also be acknowledged. First, this is a single-patient report and therefore cannot establish causal relationships or estimate disease incidence. Second, molecular characterization of the rectal cancer and hepatocellular carcinoma was not performed, limiting exploration of potential biological associations between the two malignancies. Third, although this case demonstrates the importance of pathological confirmation, the optimal timing of biopsy or surgical resection for indeterminate hepatic lesions remains individualized and should be determined according to lesion characteristics, procedural risks, and multidisciplinary consensus.

The principal clinical message of this report is that hepatic lesions identified during surveillance of colorectal cancer should not automatically be regarded as metastatic disease. In patients with chronic liver disease, particularly chronic hepatitis B infection, primary hepatocellular carcinoma should remain a major differential diagnosis. Equally important, newly developed enhancing lesions after treatment may occasionally represent benign pathological changes rather than recurrent malignancy. Comprehensive evaluation incorporating clinical history, imaging findings, serum biomarkers, and histopathological confirmation is therefore essential to achieve an accurate diagnosis and to guide optimal therapeutic management.

## 4 Patient perspective

The patient reported significant anxiety when recurrent hepatic lesions were identified during postoperative surveillance because he feared progression of his cancer. After multidisciplinary evaluation and surgical treatment, he expressed relief that a definitive diagnosis had been established through pathological examination. The patient appreciated the comprehensive management approach and remained compliant with long-term follow-up. At the latest follow-up visit, he reported satisfaction with the treatment outcomes and was encouraged by the absence of recurrent disease.

## Data Availability

The original contributions presented in the study are included in the article/supplementary material, further inquiries can be directed to the corresponding authors.
